# Interspecies interactions in dairy biofilms drive community structure and response against cleaning and disinfection

**DOI:** 10.1016/j.bioflm.2024.100195

**Published:** 2024-04-08

**Authors:** Faizan Ahmed Sadiq, Koen De Reu, Nan Yang, Mette Burmølle, Marc Heyndrickx

**Affiliations:** a Flanders Research Institute for Agriculture, Fisheries and Food (ILVO), Technology and Food Science Unit, Brusselsesteenweg 370, 9090, Melle, Belgium; b Advanced Therapies Group, School of Dentistry, Cardiff University, Cardiff, UK; c Section of Microbiology, Department of Biology, University of Copenhagen, Universitetsparken 15, 2100, Copenhagen, Denmark; d Ghent University, Department of Pathobiology, Pharmacology and Zoological Medicine, Salisburylaan 133, B-9820, Merelbeke, Belgium

**Keywords:** *Kocuria salsicia*, Cleaning & disinfection, Dairy, Biofilms, EPS, *Bacillus licheniformis*

## Abstract

Interspecies interactions within a biofilm community influence population dynamics and community structure, which in turn may affect the bacterial stress response to antimicrobials. This study was conducted to assess the impact of interactions between *Kocuria salsicia* and a three-species biofilm community (comprising *Stenotrophomonas rhizophila*, *Bacillus licheniformis*, and *Microbacterium lacticum*) on biofilm mass, the abundance of individual species, and their survival under a laboratory-scale cleaning and disinfection (C&D) regime. The presence of *K. salsicia* enhanced the cell numbers of all three species in pairwise interactions. The outcomes derived from summing up pairwise interactions did not accurately predict the bacterial population dynamics within communities of more than two species. In four-species biofilms, we observed the dominance of *S. rhizophila* and *B. licheniformis*, alongside a concurrent reduction in the cell counts of *K. salsicia* and *M. lacticum*. This pattern suggests that the underlying interactions are not purely non-transitive; instead, a more complex interplay results in the dominance of specific species. We observed that bacterial spatial organization and matrix production in different mixed-species combinations affected survival in response to C&D. Confocal microscopy analysis of spatial organization showed that *S. rhizophila* localized on the biofilm formed by *B. licheniformis* and *M. lacticum*, and *S. rhizophila* was more susceptible to C&D. Matrix production in *B. licheniformis*, evidenced by alterations in biofilm mass and by scanning electron microscopy, demonstrated its protective role against C&D, not only for this species itself, but also for neighbouring species. Our findings emphasise that various social interactions within a biofilm community not only affect bacterial population dynamics but also influence the biofilm community's response to C&D stress.

## Highlights

• Interspecies interactions in biofilms impact community dynamics and response towards antimicrobials.
• *Kocuria salsicia* enhanced cell numbers of all members of a three-species biofilm community in pair-wise interaction.
• *Stenotrophomonas rhizophila* gained growth advantages in the complex four-species biofilm community.
• Outcomes of pairwise interactions did not accurately predict complex community dynamics.
• The spatial organization in biofilms influenced survival under cleaning and disinfection (C&D) stress.

## 1 Introduction

Biofilms are structured microbial communities associated with biotic or abiotic surfaces. The microbes interact with each other and are encased within a self-produced matrix composed of extracellular polymeric substance (EPS), which regulate several functional characteristics of the biofilm's microenvironment and the residing cells [1]. The presence of undesirable microbial biofilms negatively affects several industrial and domestic processes, including, but not limited to, food and agriculture, healthcare, and wastewater sectors [2]. Biofilms in the food industry pose many food safety and quality challenges due to the biofilm-induced tolerance of microbial cells against cleaning and disinfection chemicals [3] and due to the biotransfer potential of biofilms leading to the contamination of food with not only pathogens and food spoilers, but also with their spores, enzymes and toxins [4].

Most of the bacterial biofilms in natural and industrial settings are composed of multiple and often genetically and metabolically diverse species of bacteria that interact with each other in several ways, ranging from cooperation to competition, for co-existence and better survival under environmental constraints [[5], [6], [7], [8]]. Growing evidence indicates that interactions among bacterial species in biofilms, whether through metabolic benefits or the inherent spatial structure of the biofilms, profoundly affect their ecological dynamics and response to antimicrobials [[9], [10], [11]]. Understanding the impact of interspecies interactions on individual species, as well as their collective and individual responses to antimicrobials (for instance, cleaning and disinfection chemicals: C&D), is crucial for predicting and manipulating community dynamics.

In the context of food industries, there is substantial evidence of interspecies interactions among co-localized bacteria leading to more robust biofilms. Numerous studies indicate synergistic biofilm formation involving multiple bacterial species found on food exposed surfaces across different sectors of the industry [[12], [13], [14], [15]].

In our previous research [14], we reported synergistic increases in biofilm mass within various four-species biofilm combinations. These combinations comprised bacteria that our group recovered from the surfaces of a dairy pasteurizer after C&D processes [16]. Most of the combinations contained three common bacterial species (*Stenotrophomonas rhizophila*, *Bacillus licheniformis*, and *Microbacterium lacticum*) that when together in a three-species biofilm showed a 2.65-fold increase in biofilm mass relative to the combined individual biofilm masses of the three species. When the three species were allowed to interact with a fourth species, either *Calidifontibacter indicus* or *Kocuria salsicia*. We observed 3.13-fold and 1.76-fold increases, respectively, in the four-species combinations, compared to the sum of their biofilm masses formed individually in monoculture. We conducted a thorough analysis of binary and higher-order interactions between these three species and *C. indicus*. We reported that dynamic social interactions (e.g., commensalism +/0, competition −/−, amensalism 0/-, and exploitation +/−) exist among different pairs of species within a biofilm community and are responsible for the community stability and increased biofilm mass [17].

Just as single species rarely exist in isolation, entire communities are also continually challenged and influenced by external species. In this study, we introduced *K. salsicia* (one of the dominant contaminants among others) to the three-species community and examined all possible pairwise, trio and four-species interactions. We selected *K. salsicia* as it poses a significant challenge to the food industry due to its resilience in extreme environments and its potential to cause infections [18]. This species has been isolated from biofilms in diverse food processing contexts, including dairy pasteurizers [16], milking machines [19], and meat processing facilities [20]. Moreover, *K. salsicia* has been identified in various food products, such as cheese brine [21], Turkey meat [22], and seafood [23].

The aim of this study was to uncover the interaction dynamics between the members of the three-species community and the potential impact of *K. salsicia* on community biofilm mass and individual growth of the species involved. The findings of this study bring us closer to understanding how biofilm communities are influenced by the actions of other bacterial species in an industrial setting. The impact of ecological context – specifically, the nature and dynamics of microbial interactions within a biofilm – is frequently overlooked when assessing how antimicrobials affect the susceptibility of bacteria in biofilms. Therefore, we also investigate the impact of these interspecies interactions involving *K. salsicia* on the susceptibility of the individual species in a biofilm to a laboratory scale Clean-In-Place (CIP). The insights gained from this research are essential for understanding the ecological principles that govern bacterial biofilm formation and their tolerance to C&D chemicals and perhaps for the evolution of resistance among the dairy isolates.

## 2 Materials and methods

### 2.1 Bacterial strains

The four bacterial strains used in this study were among those surviving routine C&D on the surface of a dairy pasteurizer, as reported by Maes et al [16]. They were identified and named as follows: *S. rhizophila* (B68), *B. licheniformis* (B65), *M. lacticum* (B30), and *K. salsicia* (B52). For simplicity, throughout this study, the strains are referred to as *S. rhizophila* (SR), *B. licheniformis* (BL), *M. lacticum* (ML), and *K. salsicia* (KS). However, when referencing individual species, we will use their full names. All strains were grown in a general-purpose medium (Brain-Heart-Infusion, BHI) at 30 °C.

### 2.2 Biofilm formation on polystyrene

Biofilms were grown in 96-well polystyrene microtiter plates (Coster 3596, Corning Inc., Corning, NY, USA) following the method described by Sadiq et al [14]. Briefly, bacteria were grown overnight in BHI and diluted in the same medium to the OD value of 0.05 using a Multiskan ™ FC Microplate Photometer (Thermo Fisher Scientific). An inoculum volume of 160 μl was used for monospecies biofilms, whereas equal volumes of all strains were mixed to a total volume of 160 μL in case of dual- three- and four-species biofilm combinations as reported (Ren et al., 2015). The microtiter plates were incubated for 24 h at 30 °C and the biofilms were stained with 0.1 % (w/v) crystal violet (CV) and absorbance was measured after solubilising CV with 33 % (v/v) glacial acetic acid at 595 nm (Abs595).

### 2.3 Effects of spent culture supernatants on biofilm formation

Cell-free supernatants (CFS) of each strain were generated by filtering planktonic fractions of individual species grown overnight through a 0.2-μm filter (Whatman, Germany). For the supernatant studies, biofilms were grown for 24 h, as described earlier. In each mixed-species biofilm experiment, we replaced each strain within a combination with its corresponding CFS, while keeping the total volume constant at 160 μl. This process was performed for each strain individually to assess the impact of its growth metabolites on the biofilm mass produced by the other species over a 24-h growth period. If a reduction in biofilm mass was observed due to the presence of CFS, we conducted comparative control experiments. For these controls, sterile water was added in the same volume as the replaced CFS to determine if the observed effect was due to the CFS or a result of the dilution. We also explored the impact of CFS on microbial growth by replacing live cell suspensions in BHI with CFS, which consequently altered the nutrient concentration within the growth medium – a phenomenon here described as ‘the dilution effect’.

### 2.4 Dynamics of bacterial population growth in biofilms on stainless steel (SS)

Bacterial growth dynamics of the four-species biofilm community (SR-BL-ML-KS) on SS (AISI 304 grade: 30 × 15 mm dimension) in cow's skim milk (SM) (FrieslandCampina, Belgium) and BHI growth media was assessed over the period of 24 h (at 4 h, 8 h, 12 h, 16 h, 20 h and 24 h after coincubation). Bacteria were grown overnight in BHI and diluted in the same medium to the OD value of 0.05 as described above. For experiments using SM, an equivalent volume of the overnight culture, determined by the volume necessary to reach an OD of 0.05 in BHI, was added to SM. Biofilms were allowed to form on SS coupons that were placed horizontally in 6-well microtiter plates (Coster 3516, Corning Inc., Corning, NY, USA) containing 5 mL BHI or SM. For monoculture biofilms, 5 mL were added to each well of the 6-well microtiter plate, whereas for mixed-culture biofilms, all diluted bacterial cultures were mixed to a final volume of 5 mL. The plates were incubated under static conditions at 30 °C for 24 h. For interactions in dual-species and three-species combinations, all biofilms on SS were grown only in BHI. Individual species in different combinations were counted on selective culture media plates that were developed in our laboratory (see Supplementary Material File S1). It was confirmed that the selective counting regimes had no effect on the count of bacteria when compared to their growth in monoculture on BHI agar plates.

After the required period of incubation, SS coupons were gently removed from the medium using sterile forceps and immersed in 20 mL sterile distilled water and stirred for a few seconds to remove any loosely attached cells. The coupons were then transferred to 9 mL sterile saline solution containing sterile glass beads (2 mm in diameter) and subjected to the combination of vortexing (2 min) and sonication (10min) [24] for the efficient removal of attached bacterial cells from the SS coupons. Cells were subsequently counted by serial dilution and culturing on strain-specific media plates. pH of the planktonic fractions of single and mixed-species combinations was measured with a pH meter at the 24 h sampling time.

### 2.5 Treatment of biofilms with cleaning and disinfection chemicals

Biofilms were grown on SS coupons using the previously described method. Subsequently, all monoculture and mixed-culture biofilms underwent a standard CIP regime. This regime consisted of a water rinse, followed by 1.5 % (w/v) sodium hydroxide at 60 °C for 6.45 min, another water rinse, 1.0 % nitric acid at 60 °C for 4.45 min, a further water rinse, and then disinfection with 0.01 % peracetic acid for 7.6 min, concluding with a final water rinse. Each treatment and rinse step were performed in a 6-well microtiter plate, with 5 mL of each disinfectant used per well. The CIP protocol we used at the laboratory scale was based on that designed by Bremer et al [25] and modified by consultation with a local dairy company. After C&D, the coupons were vortexed for 2 min and sonicated for 10 min to detach biofilm cells. Subsequently, they were plated on strain-specific media for counting.

### 2.6 Scanning electron microscopy (SEM)

Bacterial single and mixed-species biofilms on SS coupons grown in BHI for 24 h were subjected to SEM to observe bacterial spatial organization and biofilm matrix. After incubation, the coupons were rinsed twice with sterile water through immersion, and then the cells were fixed using 2.5 % glutaraldehyde (Sigma-Aldrich, Saint Louis, Missouri, USA) in 0.1 M sodium cacodylate (Sigma-Aldrich, Saint Louis, Missouri, USA) (pH 7.4) for >8 h. Post-fixation of biofilms was performed in 1 % osmium tetroxide in 0.1 M cacodylate for 1–2 h followed by three consecutive washings through immersion (5 min each) with 0.1 M cacodylate. Dehydration was performed in graded alcohol solution (30–100 % v/v solutions). Finally, the SS coupons were dehydrated with liquid CO_2_ in a Hitachi Model HCP-2 critical point dryer. Hitachi Model E−1010 ion sputter was used to coat the dehydrated samples with gold-palladium for 4–5 min and biofilms were imaged in Zeiss Crossbeam 540 FIB-SEM.

### 2.7 Confocal microscopy for selected dual-species combinations

Three dual-species biofilm combinations (SR-ML, SR-BL and BL-ML) were analysed by confocal microscopy to examine the bacterial spatial organization. Biofilms were grown for 24 h on plastic coupons (1 cm^2^) immersed in BHI. Fluorescence in situ hybridization (FISH) targeting 16S rRNA gene of bacteria was performed using the method described previously with modifications [26]. Biofilms on plastic coupons were washed with phosphate buffer (PBS) and fixed with 4 % paraformaldehyde in PBS (pH 7.2) at room temperature for 15 min and then incubated with lysozyme at a concentration of 1 mg/mL (w/v) at room temperature for 10 min. Samples were rinsed with PBS and dehydrated through a series of ethanol washes, containing 50 %, 75 %, and then 100 % ethanol, for 3 min each at room temperature. Following dehydration, biofilm samples were incubated with fluorochrome-labeled oligonucleotide probes at a concentration of 5 ng per 1 μL within a hybridization buffer. Three 16S rRNA-targeted FISH probes for specific detection of the three species were designed using the ProbeDealer tool based on MATLAB, as previously described [27]. The three oligo probes were synthesized commercially by Eurofins Genomics (Eurofins Scientific, France) and 5’ labeled with three different fluorochromes: Cyanine 5 (Cy5) for *S. rhizophila*, Cyanine 3 (Cy3) for *B. licheniformis*, and 6-carboxyfluorescein (FAM) for *M. lacticum*. Details of the probes can be found in Supplementary Material S1. The buffer composition was as follows: 18 % (v/v) 5 M NaCl, 2 % (v/v) 1 M Tris-HCl (pH 7.2), 30 % (v/v) formamide, 0.2 % (v/v) of a 2 % (w/v) SDS solution, 1 % (v/v) of the probes and with the remaining volume made up by water. The samples were hybridized at 46 °C for 2 h. After incubation in the hybridization mixture, samples were washed twice for 15 min at 48 °C with a washing buffer composed of 20 mM Tris-HCl (pH 7.2), 1 mM Ethylenediaminetetraacetic acid (EDTA), and 150 mM NaCl, diluted in Milli-Q water. Coupons with biofilms were air-dried and then mounted on slides using Mowiol (CAS No. 9002-89-5, Carl Roth GmbH, Germany) as the mounting medium [28,29].

Images of the biofilms formed on the coupons were captured using a confocal laser scanning microscope (LSM 800, Zeiss) with a Plan-Apochromat 63x/1.4 oil-immersion objective. Z-stacks were recorded to obtain three-dimensional (3D) images. Standard images were made with an image size of 1024 × 1024 pixels, corresponding to physical dimensions of 101.4 × 101.4 μm for each image. For each image, two separate channels were applied to detect any dual-species combination using a flexible detector (GaAsP-PMT) in the LSM 800 system. Representative 3D views of images were generated using the 3D model function in the ZEN system 3.7.

### 2.8 Statistical analyses

Each experiment was repeated three times on different occasions with three replicates in each trial. Duncan's post hoc analysis was used for statistical analysis, and it was performed using SPSS (IBM SPSS Statistics version 23.0). A paired *t*-test, calculated using GraphPad Prism 9, was used to identify potentially significant differences between bacterial cell numbers in monocultures and mixed-culture biofilms. Values of *P* ≤ 0.05 were considered statistically significant.

## 3 Results

We examined interactions within mixed-culture biofilms, focusing on two aspects: biofilm formation/biovolume on polystyrene (biofilm mass), measured in OD_595_, and cell numbers in biofilms formed on SS, quantified in log CFU/cm^2^. Observations of changes in bacterial cell numbers in both single and mixed cultures led to classifying bacterial interactions into amensalism 0/- and exploitation +/−, as previously defined by Mitri and Foster [30]. In our multispecies biofilm model composed of a panel of four species (SR-BL-ML-KS), the outcomes of interspecies interactions within various dual-species and three-species biofilms, consisting of *S. rhizophila*, *B. licheniformis*, and *M. lacticum*, have already been reported in our previous work [17]. In the present study, we report our findings on the interactions between *K. salsicia* and a three-species biofilm community when co-cultured in pairs, trios, and as a four-species community. To evaluate the effect of C&D regime, we subjected all possible dual and three-species combinations including *K. salsicia* derived from the four-species biofilm community to this treatment. This four-species combination (SR-BL-ML-KS) showed a 1.76-fold increase in biofilm mass (Fig. S1).

### 3.1 Interactions between K. salsicia and other members of the biofilm community

**Fig. 1:**
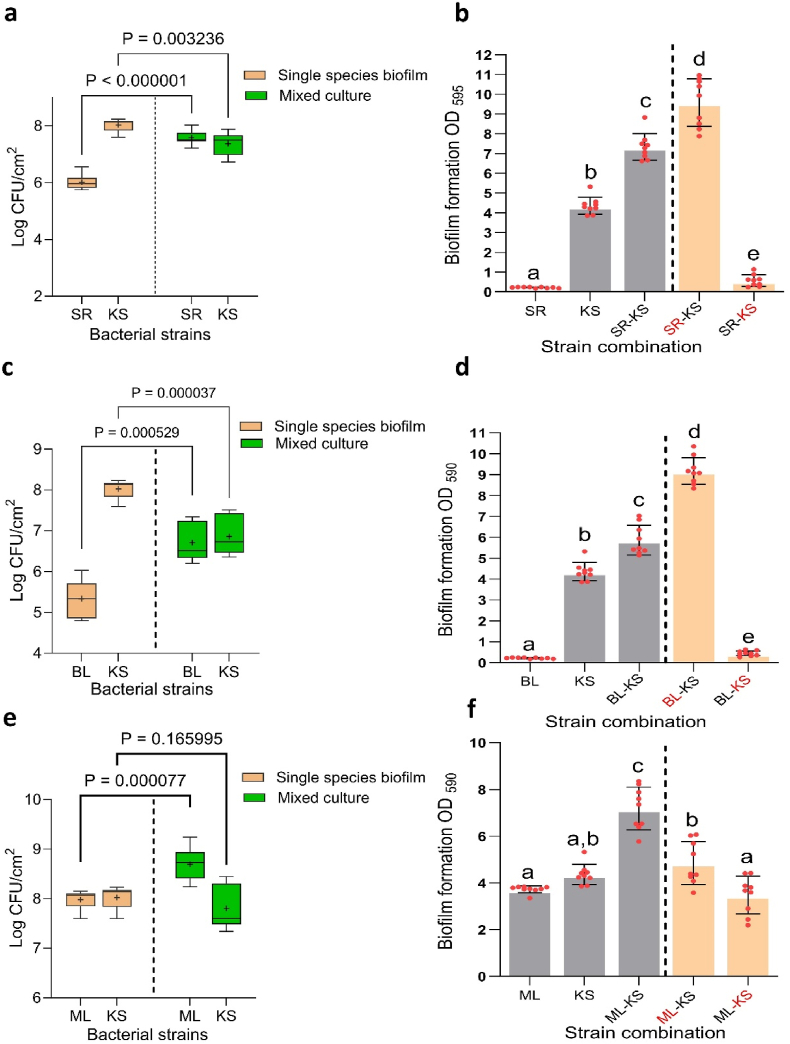
The interspecies interactions in all possible dual-species biofilm combinations among four species: *Stenotrophomonas rhizophila* (SR), *Bacillus licheniformis* (BL), *Microbacterium lacticum* (ML), and *Kocuria salsicia* (KS) are shown. The outcomes of each pairwise interaction (panels a, c, and e) are presented in terms of changes in individual cell numbers (log CFU/cm^2^) on stainless steel in both single and dual-culture biofilms after 24 h. The biofilms were cultivated on a stainless steel surface in brain-heart-infusion medium. Panels b, d, and f depict the effects of substituting each strain one at a time with its cell-free supernatant (CFS). Notably, strain numbers in red represent the presence of the strain's CFS rather than its viable form. Statistical differences in cell counts between single and co-culture biofilms for each strain were identified using a two-tailed paired *t*-test (GraphPad Prism version 9.4.1), with all corresponding two-tailed P-values displayed where significance was established at p < 0.05. Additionally, statistical differences in biofilm mass (panels b, d, and f) were evaluated using one-way analysis of variance (ANOVA), followed by Duncan's Multiple Range Test (SPSS v.23), with all significant mean differences (p < 0.05) indicated across each strain's values using letters. (For interpretation of the references to colour in this figure legend, the reader is referred to the Web version of this article.)

We first examined all possible pairwise interspecific interactions between *K. salsicia* and each other member of the four-species community (SR-KS, BL-KS, and ML-KS) on SS, as shown in Fig. 1. Our aim was to understand how *K. salsicia* affects the growth of these three species in dual-species combinations, and how these interaction outcomes may influence the biofilm mass of the four-species community. Additionally, we investigated if the CFS (derived from overnight bacterial monocultures) from one strain influenced the biofilm mass of the other species when combined.

**Fig. 2:**
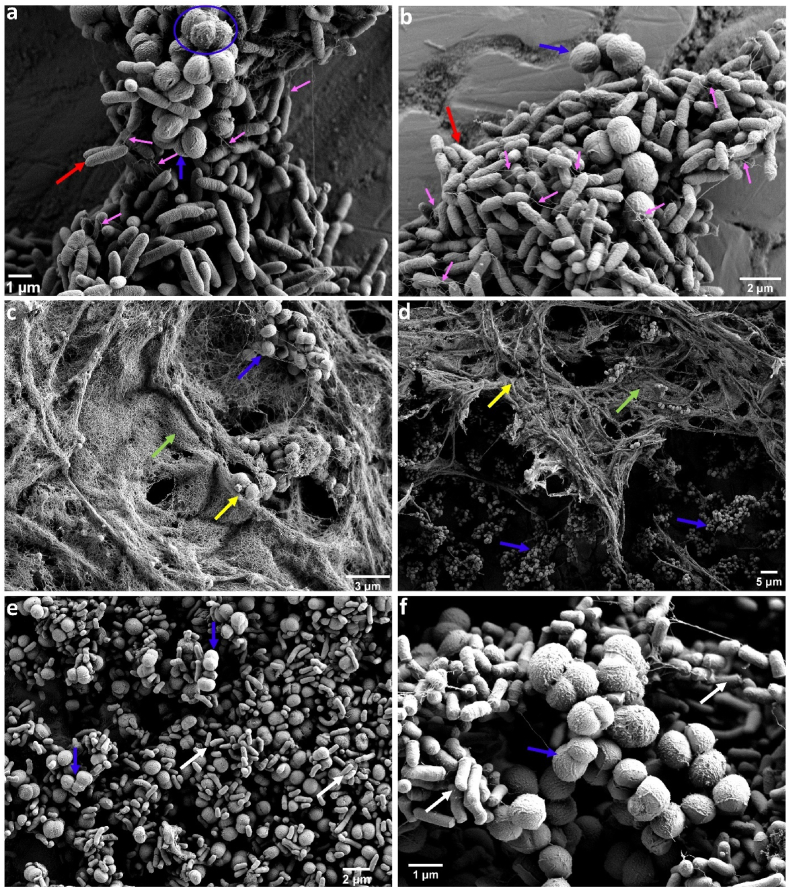
Scanning electron microscopy images of dual-species biofilms on stainless steel surfaces in brain-heart-infusion medium after 24 h. Panel (a) and (b) show a dual-species biofilm formed by *Stenotrophomonas rhizophila* and *Kocuria salsicia*. Red arrows point to *S. rhizophila* cells, and blue arrows indicate *K. salsicia* cells. The blue circle in panel (a) highlights the surface of *K. salsicia* cells surrounded by extracellular polymeric substances (EPSs). Small pink arrows indicate structures resembling flagella on *S. rhizophila* cells and interconnecting bacteria, suggesting physical interactions between cells within the biofilm. Panel (c) and (d) show a dual-species biofilm formed by *Bacillus licheniformis* and *K. salsicia* where each of these species are shown by yellow and blue arrows, respectively. EPSs are shown by green arrows. The last panel (e and f) shows a dual-species biofilm formed by *Microbacterium lacticum* and *K. salsicia*, where these two species are indicated by white and blue arrows, respectively. (For interpretation of the references to colour in this figure legend, the reader is referred to the Web version of this article.)

In all three dual-species interactions, *K. salsicia* enhanced the growth of the other species involved. *S. rhizophila* and *B. licheniformis* exploited *K. salsicia* (±), resulting in a significant increase in their own growth while simultaneously inhibiting the growth of *K. salsicia* compared to its monoculture biofilm growth. The biofilm mass of the co-cultures SR-KS and BL-KS increased by 1.6 and 1.3-fold, respectively, compared to the combined biofilm masses of their individual monocultures (Fig. 1a and c, respectively). The SEM images of biofilms on SS, shown in Fig. 2 (panels a & b), show the abundance of *S. rhizophila* in the SR-KS combination from two different sections of the SS surface. Additionally, in the BL-KS combination, *B. licheniformis* cells induced matrix production, which completely covered the surface area (Fig. 2: c & d). It remained unclear whether the increase in biofilm mass was due to growth leading to higher cell numbers of *S. rhizophila* and *B. licheniformis* in the respective combination, or if it was a result of enhanced matrix production by these species or by *K. salsicia*. The CFS from *S. rhizophila* (Fig. 1b) and *B. licheniformis* (Fig. 1d) increased the biofilm mass of *K. salsicia* by 2.2-fold and 2.1-fold, respectively, compared to its biofilm mass in monoculture. Interestingly, the biofilm mass in these combinations was significantly higher than in the SR-KS and BL-KS combinations, where viable cultures of both species were used instead of CFS. On the other hand, CFS of *K. salsicia* significantly increased the biofilm mass of both *S. rhizophila* (Fig. 1b) and *B. licheniformis* (Fig. 1d) by 2.6-fold and 2-fold, respectively, compared to their biofilm masses in monoculture. These observations suggest that matrix production by both *K. salsicia* and *B. licheniformis* might have contributed to the overall biofilm mass, in addition to the higher cell numbers of *B. licheniformis* and *S. rhizophila*. The SEM image of the dual-species biofilm SR-KS reveals EPS production confined to the cells of *K. salsicia* on its surface (Fig. 2a). This was not the case for EPS production in the BL-KS biofilm, as observed in the SEM image, where EPS production was enhanced due to *B. licheniformis* cells, a characteristic typical of this species in many mixed-culture biofilms, presumably as a stress response mechanism (Fig. 2), as observed in our previous trials [17].

The cocultivation of *K. salsicia* and *M. lacticum* also led to increased cell numbers of *M. lacticum* (0.7 log CFU/cm^2^) in the dual-species biofilm, compared to its monoculture biofilm (Fig. 1e). However, the cell counts of *K. salsicia* remained unchanged and thus this interaction was termed “amensalism (0/-)”. SEM images show abundance of both species and cell-cell association between *K. salsicia* and *M. lacticum* within the dual-species biofilm (Fig. 2, panels e and f). Interestingly, CFS from either species did not seem to affect the other's growth, as no statistically significant changes in biofilm mass were observed.

### 3.2 Higher-order interactions of K. salsicia and the other species

**Fig. 3:**
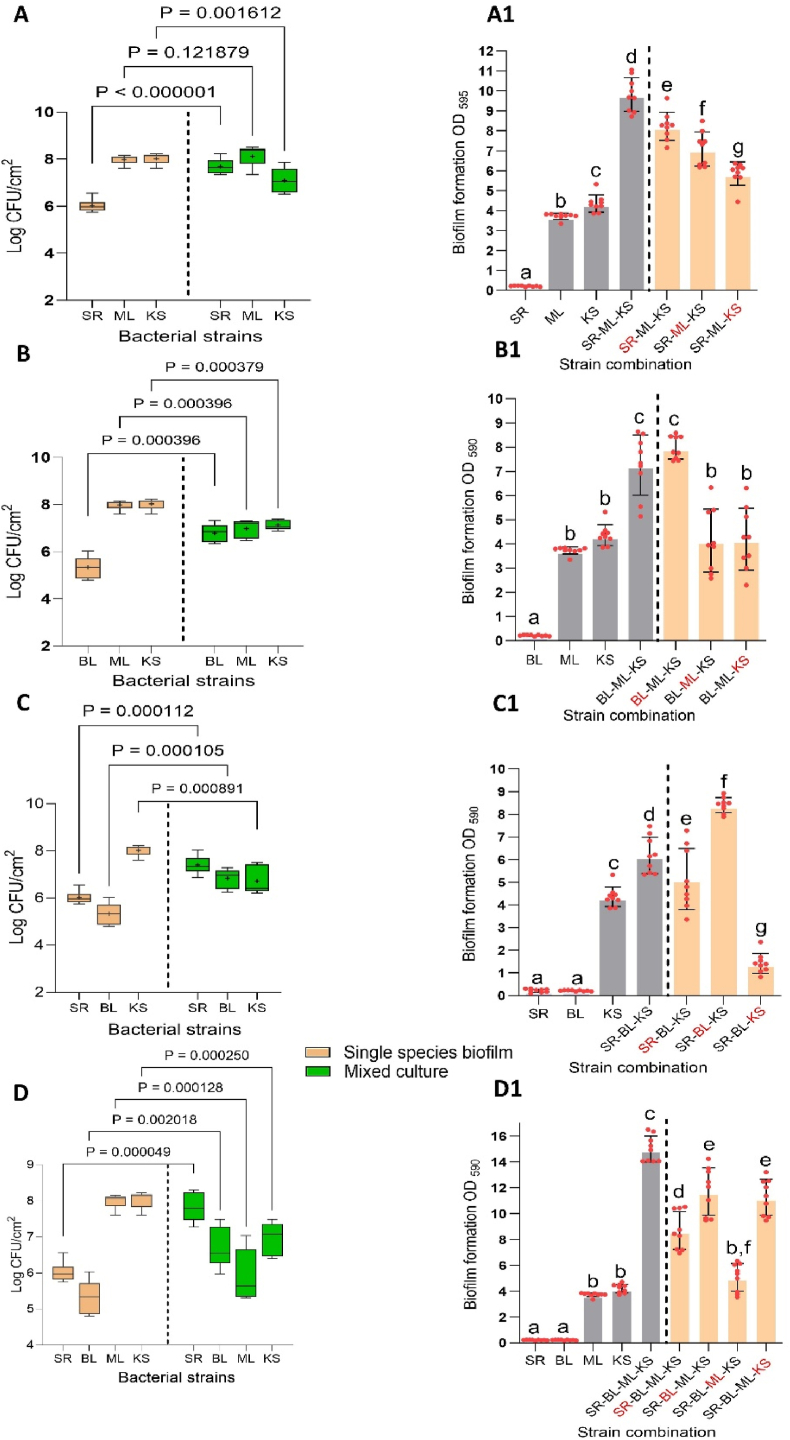
The interspecies interactions in all potential three-species biofilm combinations among four species: *Stenotrophomonas rhizophila* (SR), *Bacillus licheniformis* (BL), *Microbacterium lacticum* (ML), and *Kocuria salsicia* (KS) are shown. The outcomes of each pairwise interaction (panels A, B, C and D) are presented in terms of changes in individual cell numbers (log CFU/cm^2^) on stainless steel in both single and three-species biofilms after 24 h. The biofilms were cultivated on a stainless steel surface in brain-heart-infusion medium. Panels b, d, and f depict the effects of substituting each strain one at a time with its cell-free supernatant (CFS). Notably, strain numbers in red represent the presence of the strain's CFS rather than its viable form. Statistical differences in cell counts between single and co-culture biofilms for each strain were assessed using a two-tailed paired *t*-test (GraphPad Prism version 9.4.1), with all corresponding two-tailed P-values displayed where significance was established at p < 0.05. Additionally, statistical differences in biofilm mass (panels A1, B1, C1 and D1) were evaluated using one-way analysis of variance (ANOVA), followed by Duncan's Multiple Range Test (SPSS v.23), with all significant mean differences (p < 0.05) indicated across each strain's values using letters. (For interpretation of the references to colour in this figure legend, the reader is referred to the Web version of this article.)

After studying the interaction of *K. salsicia* with individual species, it was incorporated as the third species together with the two other species in the biofilm community panel (SR-ML, BL-ML, and SR-BL) (Fig. 3: A-C1). Additionally, *K. salsicia* was added as the fourth species together with the three other species in the biofilm community panel (SR-BL-ML) (Fig. 3D-D1). Subsequently, we assessed the changes in biofilm mass and cell numbers of *K. salsicia* and of the other two or three species (Table 1). In this study, our focus is primarily on examining the interactions of *K. salsicia* with the biofilm community panel comprising *S. rhizophila*, *B. licheniformis*, and *M. lacticum* (SR-BL-ML). It is important to note that the results related to biofilm biomass and bacterial cell counts, arising from interactions among *S. rhizophila*, *B. licheniformis*, and *M. lacticum*, are sourced from our previous publication [17]. This previously published data, included in Table 1 of the current manuscript is for reference and to facilitate a comprehensive understanding of the biofilm dynamics. In some instances, we have also made direct comparisons between the data obtained in this experiment and data from another experimental batch, as shown in Table 1. The trend of bacterial population dynamics remained consistent in the three-species communities compared to their growth in dual-species combinations, as shown in Table 1. For instance, the cell numbers of *S. rhizophila* and *B. licheniformis* significantly increased in the SR-ML-KS combination (Fig. 3:A), while there was a corresponding decrease in the cell number of *K. salsicia*, consistent with the observed interaction in SR-KS and BL-KS, respectively (Fig. 1, a and c, respectively). The cumulative biofilm mass of the three-species biofilms did not show additive effects compared to sum of the biofilm masses of the pair and *K. salsicia* when cultured separately, except for the SR-BL-KS combination. For example, the biofilm mass for the BL-ML-KS combination (estimated from the CV staining and absorbance) resulted in an OD value of 7.3 (Fig. 3:B1). This contrasts with the expected cumulative biofilm mass of 10.7 (OD_595_), calculated by adding up the individual masses of the dual-species combination BL-ML (6.5, Table 1) and *K. salsicia* (4.2) in isolation. This finding was ascribed to changes in bacterial cell counts, stemming from growth effects in more complex communities (refer to Table 1 for details). It shows that direct linear extrapolations from low to high complexity communities are not valid. In the ML-KS pair (Fig. 1:e), cell numbers of *M. lacticum* increased by 0.71 log/CFU/cm^2^ due to *K. salsicia*, while in the ML-BL pair, it decreased by 0.9 log/CFU/cm^2^ due to *B. licheniformis* (Table 1). However, in the BL-ML-KS community panel, the cell number of *M. lacticum* decreased by approximately 1 log/CFU/cm^2^ (Fig. 3:B). This suggests that while *K. salsicia* promotes the growth of *M. lacticum*, this benefit is offset in the three-species community by *B. licheniformis* which is increased by 1.44 log/CFU/cm^2^. In addition, the negative effect of *B. licheniformis* and *S. rhizophila* on the cell count of *K. salsicia* in the three-species combination (SR-BL-KS) (Fig. 3:C) was not the sum of the growth-inhibiting effects observed in the respective dual-species combinations of BL-KS and SR-KS. This indicates that the fate of a given bacterial species in complex communities depends on the interactions with and between the other species, which either supports or suppresses its growth.

**Table 1:** A comparison of biofilm mass on polystyrene (OD_595_) and cell numbers on stainless steel (log CFU/cm^2^) between different dual-, three-, and four-species combinations to assess the effect of adding different species on the interaction of other species present. SR: *Stenotrophomonas rhizophila*; BL: *Bacillus licheniformis*; ML: *Microbacterium lacticum* and KS: *Kocuria salsicia*. Biofilm biovolume and individual cell counts for *S. rhizophila*, *B. licheniformis*, and *M. lacticum* in various mixed-culture biofilms were derived from another experimental batch. This data has been previously published [17].

Combination	Cell count on Stainless steel (log CFU/cm^2^)					
	Biofilm mass OD_595_ (polystyrene)	*S. rhizophila*	*B. licheniformis*	*M. lacticum*	*K. salsicia*	Reference
**SR-ML-KS**	9.81	↑ 1.68	–	↑ 0.13	↓ 0.94	This study
SR-ML	5.71	↑ 2.0	–	↑ 0.19		[17]
SR-KS	7.32	↑ 1.57	–		↓ 0.63	This study
ML-KS	7.18	–		↑ 0.71	↓ 0.22	This study
**BL-ML-KS**	7.25	–	↑ 1.44	↓ 1.0	↓ 0.91	This study
BL-ML	6.45	–	↑ 1.41	↓ 0.90	–	[17]
BL-KS	5.87	–	↑ 1.37	–	↓ 1.16	This study
ML-KS	7.18	–	–	↑ 0.71	↓ 0.22	This study
**SR-BL-KS**	6.18	↑ 1.39	↑ 1.50	–	↓ 1.30	This study
SR-BL	1.23	↓ 0.05	↑ 0.08	–	–	[17]
SR-KS	7.32	↑ 1.57	–	–	↓ 0.63	This study
BL-KS	5.87	–	↑ 1.37	–	↓ 1.16	This study
**SR-BL-ML-KS**	14.99	↑ 1.95	↑ 1.34	↓ 2.1	↓ 1.10	This study
SR-BL-ML	10.99	↑ 1.94	↑ 1.73	↓ 1.09	–	[17]

**Fig. 4:**
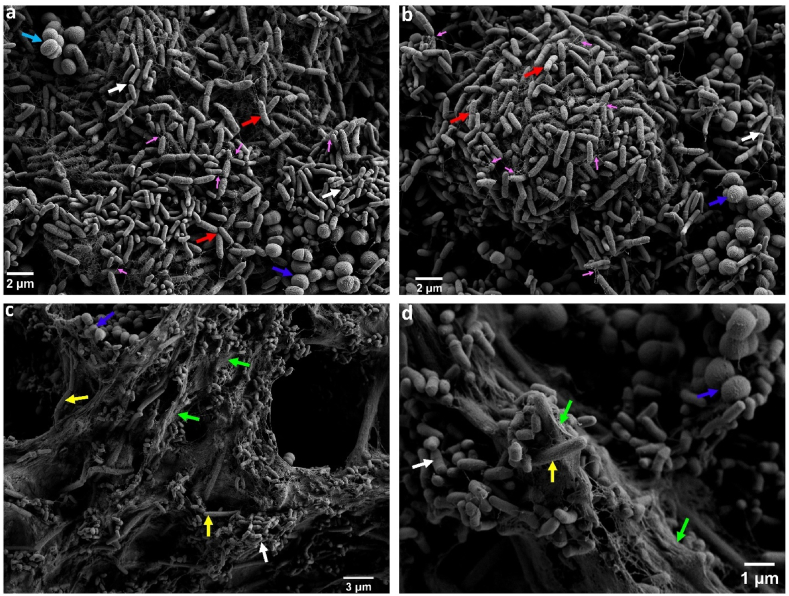
Scanning electron microscopy images of three-species biofilms. Panel (a) and (b) indicate biofilms formed by *Stenotrophomonas rhizophila*, *Microbacterium lacticum*, and *Kocuria salsicia* on a stainless steel surface in brain-heart-infusion medium after 24 h. In these panels, *S. rhizophila*, *M. lacticum*, and *K. salsicia* are indicated by red, white, and blue arrows, respectively. Pink arrows in panel (a) and (b) indicate structures resembling flagella protruding from *S. rhizophila* cells and interconnecting bacteria, suggesting physical interactions between cells within the biofilm. The panel (b) and (c) indicate biofilms formed by *Bacillus licheniformis*, *M. lacticum*, and *K. salsicia*, with these species represented by yellow, white, and blue arrows, respectively. Extracellular polymeric substances (EPS) are highlighted with green arrows. (For interpretation of the references to colour in this figure legend, the reader is referred to the Web version of this article.)

**Fig. 5:**
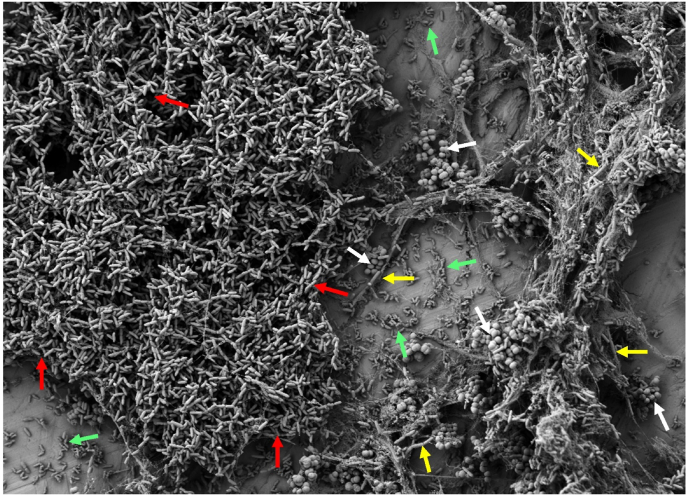
Scanning electron microscopy images of a four-species biofilm formed by *Stenotrophomonas rhizophila*, *Bacillus licheniformis*, *Microbacterium lacticum*, and *Kocuria salsicia* on a stainless steel surface in brain-heart-infusion medium after 24 h. *S. rhizophila*, *B. licheniformis*, *M. lacticum*, and *K. salsicia* are indicated by red, yellow, blue, and white arrows, respectively. (For interpretation of the references to colour in this figure legend, the reader is referred to the Web version of this article.)

The presence of *S. rhizophila* is visualised in SEM images of all dual, three-species and four-species combinations involving this species (Fig. 2, Fig. 4, Fig. 5). Fig. 4 (a and b) shows structures similar to flagella extending from the cell surface of *S. rhizophila* cells, linking the cells. Such structures have been observed in *Stenotrophomonas maltophilia* biofilms, where they facilitate connections to other bacteria [31]. Fig. 4 (c and d) demonstrates significant EPS production by *B. licheniformis* cells when co-cultured with *M. lacticum* and *K. salsicia*. This finding aligns with similar observations noted in the BL-ML [17] and BL-KS (this study) combinations.

We observed higher-order interactions when evaluating the effect of CFS from one species on the growth of another species in pairs. For instance, CFS of *S. rhizophila* stimulated the biofilm mass production by combination ML-KS (∼1.2-fold) (Fig. 1, Fig. 3f). Meanwhile, the CFS of *B. licheniformis* increased biofilm mass production in *S. rhizophila* and *K. salsicia* in combination SR-KS (1.2-fold) (Fig. 1, Fig. 3b). An increase in biofilm mass may be attributed to the EPS production by *K. salsicia* when exposed to the CFS from *S. rhizophila* and *B. licheniformis*. This observation is corroborated by the pronounced increase in biofilm mass of *K. salsicia* in the presence of CFS from either of these species.

The comparison of cell counts among all species within the four-species biofilm and their respective monoculture biofilms is illustrated in Fig. 3D. To validate the impact of individual species on the observed synergistic effects, both in their viable form and CFS fractions, cells of each of the species in the four-species biofilm was systematically replaced with its corresponding CFS in the four-species biofilm community, as shown in Fig. 3(D1). The resulting biofilm mass of the community, which consisted of cells of three strains along with the CFS of the fourth strain, was then compared to the biofilm mass of the three-species biofilm community (Table 1). In three-species biofilm combination (SR-BL-KS), the impact of the presence of *M. lacticum* in viable form is illustrated by the fact that replacing viable cells with its CFS reduced the biofilm mass from 15 to 5.1 (Fig. 3 D1). In contrast, CFS from *B. licheniformis* and *S. rhizophila* significantly increased the biofilm mass of combinations BL-ML-KS and SR-ML-KS, respectively, by 17 and 47 % respectively, compared to live BL and SR cells, which could be as a result of matrix production by *K. salsicia* in the presence of CFS of the either species as observed in pair-wise interactions (Fig. 3 D1). CFS from *K. salsicia* did not appear to significantly affect the biofilm mass of the remaining species (Fig. 3 D1).

### 3.3 Growth dynamics in the four-species biofilm

We quantified the relative abundance and growth dynamics of each bacterial species within the four-species biofilm community, both in planktonic and biofilm fractions on SS. This was done at six different time points over a 24-h period (at 4, 8, 12, 16, 20, and 24 h) in BHI and SM media. The aim was to observe how species diversity changes in different media and in biofilms versus planktonic fractions over 24 h (Fig. S3). All species exhibited differing growth dynamics in both planktonic and biofilm fractions over a 24-h period. We included SM as a growth medium alongside BHI to compare bacterial growth dynamics between the two media. The growth in the two media was notably similar. However, the bacterial abundances differed between planktonic and biofilm fractions. In the mixed-species biofilm formed in SM, *M. lacticum* grew to higher cell numbers (Fig. S3). *M. lacticum* cell numbers decreased after 20 h in SM, in contrast to 12 h in BHI. In the biofilm fractions of the four-species community grown in both SM and BHI media, *K. salsicia* showed significantly different cell numbers. After a 24-h incubation period, approximately 3.5 log CFU/cm^2^ of *K. salsicia* cells were observed in the planktonic fractions cultured in both SM and BHI media. In contrast, the mixed-species biofilm fractions cultured in SM and BHI exhibited a higher cell density of ∼7 log CFU/cm^2^.

Higher cell numbers for both *S. rhizophila* and *B. licheniformis* were observed in mixed-species planktonic fractions compared to the mixed-species biofilm communities. In the four-species biofilm, the cell count of *S. rhizophila* markedly increased from 5.6 log CFU/cm^2^ at 4 h to 7.8 log CFU/cm^2^. Consequently, it emerged as the predominant species, representing approximately 79 % of bacterial cells present, a significant increase from merely 10 % at the 4-h time point. Similarly, *B. licheniformis* increased from 0.98 % (4.58 log CFU/cm^2^) at the 4-h time point to approximately 8 % (7 log CFU/cm^2^) at the 24-h time point. On the other hand, both *M. lacticum* and *K. salsicia* exhibited a decrease in abundance from 57.8 % to 30.7 %, respectively, at the 4-h time point to 2.15 % and 11.1 %, respectively, at the 24-h time point.

The numerical data related to biofilm mass based on CV staining presented in Fig. 1, Fig. 3 are visually supported by the images of the crystal violet staining assays, provided in Fig. S2.

### 3.4 pH of the planktonic fractions of all combinations

The pH in the planktonic fractions of various mixed-species biofilm combinations were quantitatively assessed and compared with the pH of monoculture planktonic fractions after a 24-h growth period, as shown in Table 2. Changes in pH would be linked with the proportions of each species as *M. lacticum* and *K. salsicia* lowered the pH of BHI to around 6, whereas, the growth of both *S. rhizophila* and *B. licheniformis* raised the pH to above 8*.* The pH of the planktonic fractions in the BL-ML-KS (pH 7.5) and SR-BL-ML-KS (pH 8.5) combinations showed a pH more conducive to the growth of *S. rhizophila* and *B. licheniformis*, but less conducive for *K. salsicia*. This is consistent with the cell counts retrieved from these combinations.

**Table 2:** The pH values of the planktonic fraction of pure and different combinations of strains after 24 h. SR = *Stenotrophomonas rhizophila*, BL= *Bacillus licheniformis*, ML = *Microbacterium lacticum* and KS= *Kocuria salsicia*. The values represent mean values ± standard deviation obtained in three independent experiments.

Name	pH of the planktonic fraction ±SD
SR	8.26 ± 0.06
BL	8.04 ± 0.04
ML	6.00 ± 0.03
KS	6.5 ± 0.37
SR + KS	8.02 ± 0.05
BL + KS	7.60 ± 0.14
ML + KS	6.04 ± 0.06
SR + ML + KS	7.18 ± 0.13
BL + ML + KS	7.54 ± 0.10
SR + BL + ML + KS	8.52 ± 0.14

### 3.5 Response of bacterial species towards C&D chemicals

**Fig. 6:**
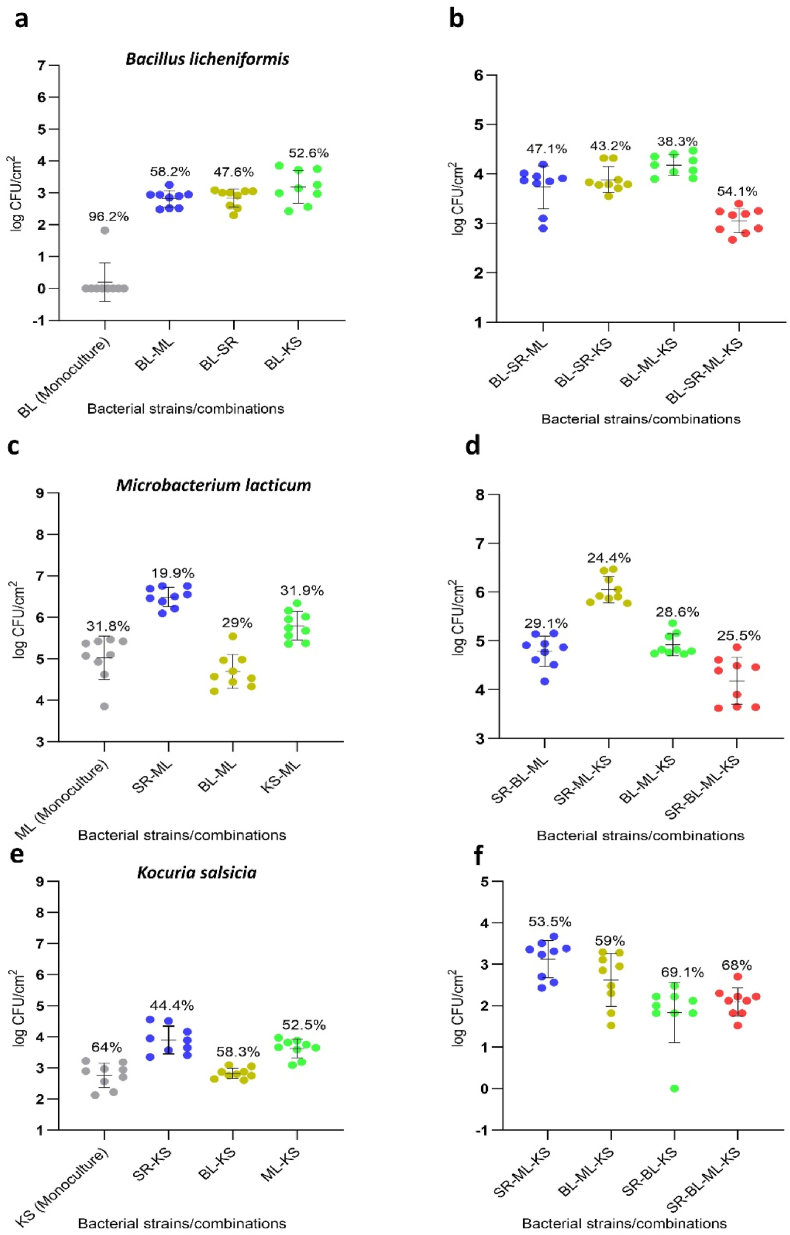
Survival of *Bacillus licheniformis* (BL), *Microbacterium lacticum* (ML), and *Kocuria salsicia* (KS) following laboratory-scale cleaning and disinfection (C&D) treatment is shown. Panels a, c, and e show the numbers of surviving cells of *B. licheniformis*, *M. lacticum*, and *K. salsicia*, respectively, in monoculture and different dual-species biofilm combinations after C&D, expressed in log CFU/cm^2^ on stainless steel. Panels b, d, and f show the numbers of surviving cells of *B. licheniformis*, *M. lacticum*, and *K. salsicia*, respectively, in various multispecies biofilm combinations following C&D, also in log CFU/cm^2^ on stainless steel. Values (in percentages) on top of the datapoints represent the reduction in the number of each bacterial cell from their initial count in monoculture or any specific mixed-culture, expressed as a percentage.

The response of bacterial species to C&D chemicals aligned with their inherent resistance capabilities, their interspecies interactions and spatial organization. Fig. 6 shows the bacterial counts (log CFU/cm^2^) of *B. licheniformis*, *M. lacticum*, and *K. salsicia* in various mixed-species biofilm combinations after C&D. Percentages above data points indicate the reduction in bacterial counts relative to the initial cell numbers of each bacterial species before C&D. It should be noted that bacterial cell counts in different mixed-culture biofilms varied depending on the type of interactions. Information related to the initial bacterial cell numbers for each mixed biofilm combination is listed in Table 3. The figure compares the number of bacterial cells that survived post-C&D to their initial cell numbers. These initial cell numbers were determined in a separate experimental batch, the results of which have been previously published [17].

**Table 3:** This table presents the cell counts of each bacterial species, originally present due to paired interactions, before cleaning and disinfection (C&D) treatment. The reduction in cell counts (%) after C&D reported in Fig. 6 are based on these cell counts. The species are abbreviated as SR for *Stenotrophomonas rhizophila*, BL for *Bacillus licheniformis*, ML for *Microbacterium lacticum*, and KS for *Kocuria salsicia*. The data sources include the current study and cell count information for each pair from our previous study [17].

Name of the combination	SR	BL	ML	KS	Source
BL-ML	–	6.75	7.08	–	[17]
BL-SR	6	5.42	–	–	[17]
BL-KS	–	6.71	–	6.86	This study
ML-SR	7.86	–	8.17	–	[17]
ML-KS	–	–	8.69	7.8	This study
KS-SR	7.57	–	–	7.37	This study
BL-SR-ML	8.31	7.06	6.88	–	[17]
BL-SR-KS	7.4	6.84	–	6.72	[17]
BL-ML-KS	–	6.77	6.98	7.12	This study
BL-SR-ML-KS	7.97	6.67	5.89	6.92	This study

**Fig. 7:**
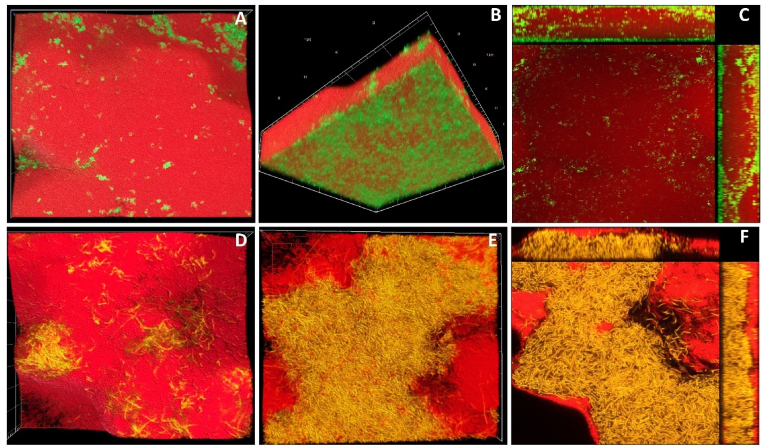
Confocal laser scanning microscopy images of dual-species biofilms on plastic coupons in brain-heart-infusion medium after 24 h. Panels (A) and (B) show top and bottom views of a biofilm composed of *Microbacterium lacticum* (green) and *Stenotrophomonas rhizophila* (red), with *M. lacticum* predominantly forming a basal layer beneath an overlying *S. rhizophila* biofilm. Panel (C) presents a cross-sectional view of this biofilm, highlighting the layered structure. Panels (D), (E), and (F) show the top, bottom, and cross-sectional views, respectively, of a biofilm formed by *Bacillus licheniformis* (yellow) and *S. rhizophila* (red), illustrating layered spatial organization. (For interpretation of the references to colour in this figure legend, the reader is referred to the Web version of this article.)

*S. rhizophila* did not survive the C&D process in neither monoculture nor mixed-culture biofilms, which is why data on this species is not presented in Fig. 6*.* Yet, in certain dual-species combinations, the presence of *S. rhizophila* resulted in increased survival rates for the other species. In co-culture experiments, *S. rhizophila* conferred a protective effect on *B. licheniformis* (BL-SR), *M. lacticum* (ML-SR), and *K. salsicia* (KS-SR) during C&D. The reduction in cell numbers following C&D was significantly lower in co-cultures with *S. rhizophila* compared to monocultures: *B. licheniformis* (47.6 % vs. 96.2 %), *M. lacticum* (19.9 % vs. 31.8 %), and *K. salsicia* (44.4 % vs. 64 %). These findings highlight the importance of the presence of *S. rhizophila* in enhancing the survival of other bacterial species during C&D. This protective effect could be related to the bacterial spatial organization within different biofilm combinations. Confocal laser scanning microscopy (CLSM) images confirmed the presence of *S. rhizophila* on top of other bacterial cells, such as *M. lacticum* (panels A, B, and C of Fig. 7) and *B. licheniformis* (panels C, D, and E of Fig. 7). Different cross-sectional layers of the dual-species biofilm comprising *S. rhizophila* and *M. lacticum* are shown in Fig. S4. Fig. S5 shows the layered organization of the biofilm, highlighting the dominance of *S. rhizophila* in the top layer above *B. licheniformis*. This suggests a competitive edge for *S. rhizophila*, potentially outcompeting B. licheniformis for space within the biofilm structure.

The dual-species CLSM images of *B. licheniformis* and *M. lacticum* biofilms on SS and plastic surfaces showed a thorough mixing of the species without any specific spatial arrangement (Fig. S6). The survival rate of certain species diminished in complex biofilms. For instance, *B. licheniformis* survived better in a three-species combination with *S. rhizophila* and *K. salsicia* (BL-SR-KS), compared to its monospecies biofilm survival, yet it had the lowest survival when *M. lacticum* was added to form a four-species biofilm (Fig. 6b). The survival rate of *B. licheniformis* also decreased in the four-species biofilm community compared to various dual-species combinations. *K. salsicia* exhibited a comparable reduction in its count (log CFU/cm^2^) both in monoculture biofilm (with a 64 % reduction) and within the four-species combination (with a 68 % reduction). However, it remained relatively more abundant in the monoculture biofilm. It should be noted that the survival rate of each species was dependent on the specific biofilm combination. Furthermore, survival in various combinations was not merely an additive effect; rather, it was influenced by the presence or absence of other species within more complex communities.

## 4 Discussion

Within the complex milieu of biofilm communities, interspecies interactions shape the overall dynamics and success of the consortium. In our previous work [17], we observed synergistic interactions within a three-species biofilm community of dairy origin, consisting of *S. rhizophila*, *B. licheniformis*, and *M. lacticum*. When co-cultured, these bacteria produced biofilm mass 2.7-fold greater than the sum of their individual monoculture biofilms [17]. The addition of *K. salsicia* as a fourth species resulted in a further 1.8-fold increase in biofilm mass. In finding the possible explanation behind the synergistic increase in biofilm mass of the four-species biofilm community, we observed that all species in the consortium were indispensable for the observed synergy. However, not all species reaped growth benefits from being part of the community. All species within the three-species biofilm (SR-BL-ML) showed enhanced growth when *K. salsicia* was added. However, while *K. salsicia* experienced reduced growth in this four-species biofilm consortium, it was more abundant within the mixed-species biofilm than in a mixed-species planktonic fraction. This observation was corroborated by *K. salsicia* cell counts, monitored every 4 h over a 24-h period.

These observations mirror the ecological concept of “keystone species” in larger ecosystems, where certain species have a disproportionately large influence on their environment and community structure [17,32]. However, unlike traditional keystone species which often benefit from their role, *K. salsicia* appears to be paying a fitness/growth cost for its central role in the biofilm ecosystem. It remains uncertain whether *K. salsicia* supplies specific growth factors or nutrients to its co-existing species, leading to its potential exploitation. The interspecific interactions observed between *S. rhizophila*, *B. licheniformis*, and *K. salsicia* are noteworthy from an ecological perspective. The increase in biofilm mass observed in the dual-species consortia (SR-KS and BL-KS) cannot be attributed merely to an increase in the cell numbers of *S. rhizophila* and *B. licheniformis*. Rather, it suggests a physiological response of *K. salsicia* and *B. licheniformis* to potential competitive or antagonistic factors, leading to induced EPS production. This preposition is further substantiated by the pronounced increase in the biofilm biomass of *K. salsicia* and *B. licheniformis* upon exposure to the CFS from each other. Bacteria have been reported to induce biofilm matrix pathways in response to stress induced by competitive species [33]. Oliveira et al [34] proposed that biofilm formation is not just a passive growth mode but a dynamically regulated response to ecological competition. Marvasi et al [35] defined EPS as structural molecules released in response to physiological stress encountered in the natural environment, characteristic of the genus *Bacillus*. It is important to clarify that the increased EPS production, evident in the SEM images, is predominantly attributable to *B. licheniformis*. This observation aligns with our previous findings [17] and is a typical stress response strategy of this species.

Bacterial interactions are frequently mediated by environmental modifications, such as pH changes, resulting from various metabolic activities due to resource utilization and metabolite excretion [36,37]. A reduced cell number of *K. salsicia* could be related to unfavourable pH for the growth of this species. A recent study by Ref. [38] also demonstrated that bacterial activities can cause the pH of their environment to deviate from neutrality. This alteration in pH creates feedback loops that impact not only the bacteria's own growth and viability but also affect the dynamics of other species in the ecosystem through growth facilitation, competitive exclusion, or extinction. The effect of the pH on the interactions among the species in question could not be accurately predicted without the knowledge of their optimum pH for growth.

Our results corroborate the findings of Baichman-Kass et al [39], who assayed thousands of two, three, and four-species bacterial communities and demonstrated that competitive interactions between culturable bacteria were mainly non-additive. Specifically, the observed negative effects on *K. salsicia* in dual-species combinations, as compared to three-species combinations, indicate that these effects are not additive in a three-species community. Instead, they are dominated by the most pronounced effects of pair-wise interactions. Non-additive effects were also noted when the biofilm masses of all possible pairs in a three-species community panel were compared to the biofilm mass of the entire three-species community. Two species may not necessarily interact in the same way within more complex communities, as the presence of other species can affect growth conditions and interactions [40,41]. Thus, higher-order interactions are non-linear and cannot be predicted merely by summing up the effects of all possible pairwise interactions. Instead, the outcomes related to community biofilm biomass and growth dynamics are contingent upon the complexity of the community and the nature of inherent higher-order interactions - the modulation of a pairwise interaction by a third species [42]. Ishizawa et al [43] introduced a simplified model to understand higher-order ecological interactions, demonstrating that predictions of community dynamics based on three-species combinations are more accurate than those based on pairwise interactions or more complex community structures. Our observations reveal that while growth dynamics in higher-order communities mirror those in pairwise interactions, this reflection is not exact, and these interactions are not entirely predictive. In multi-species communities, a species that enhances the growth of another in a pairwise setup may itself be outcompeted/exploited in a three- or four-species biofilm community. Consequently, its ability to confer a growth advantage is diminished, highlighting the complex nature of interspecies dynamics. Aguilar-Salinas and Olmedo-Álvarez [44], using a three-species bacterial model, also reported that outcomes of paired interactions do not predict community dynamics. They observed that emergent features of ecological communities often attenuate several antagonistic functions seen in pairwise interactions.

In the present study, the four-species biofilm community favoured the growth of *S. rhizophila* and *B. licheniformis*, while growth of *M. lacticum* and *K. salsicia* was reduced. This dynamic did not reflect a balanced, non-transitive growth pattern typical of the ‘rock-paper-scissors' analogy. Instead, the growth facilitation of *S. rhizophila* and *B. licheniformis* suppressed the growth of both *M. lacticum* and *K. salsicia*. The introduction of higher-order interactions, such as antagonists to *S. rhizophila* or *B. licheniformis*, could yield different results. This supports the notion that interspecies interactions are context-dependent, varying with the species involved and influenced by environmental conditions, as previously reported [45].

In many microbial communities, biofilm formation is influenced by cell-to-cell signalling and other secreted factors found in CFS [46]. They can promote or inhibit the growth and biofilm formation of other microbes, leading to intricate interaction dynamics. The observed increase in biofilm mass for the two interacting pairs of species (ML-KS and SR-KS) in the presence of CFS from *S. rhizophila* and *B. licheniformis*, respectively, may be partly due to induced EPS production in *K. salsicia*. This induction could be a response to the CFS from *S. rhizophila* and *B. licheniformis*, as evidenced in dual-species combinations involving *K. salsicia* with either of these species.

Bacterial communities survive in complex and variable environments by using different cooperative strategies of which cooperation is one [47]. Since the C&D chemicals were applied to biofilms that had already grown for 24 h, their effect on bacterial survival could be related to either the spatial organization of the bacteria within a specific combination and/or to EPS production. The spatial arrangement of different bacteria within a community has been shown to influence cooperative and competitive cell-cell interactions [48]. Multispecies biofilm architecture has also been linked to bacterial community dynamics and stress response against phages [49]. The bacterial response to C&D in both monoculture and different mixed-culture biofilms linked to specific biofilm combinations which could be related to bacterial spatial organization and different EPS production. However, we demonstrated that spatial organization also affects bacterial survival in response to C&D treatment*. S. rhizophila* did not survive C&D treatment in monoculture or in any mixed-culture biofilm; however, it provided protection to other species from C&D treatment. This may be because it forms biofilms on the surface of other co-existing species and becomes an abundant community member in all combinations. This could enhance nutrient and electron acceptor availability to *S. rhizophila* and thus confer a growth advantage in the absence of inhibitory conditions; however, it becomes a disadvantage when the biofilm is subjected to C&D chemicals, as they target the biofilm top layers first and have less effect on the cells beneath the top layers.

Our previous work demonstrated EPS production by *B. licheniformis* when co-cultured with *M. lacticum* and S. *rhizophila* [17]. From this study, SEM images confirm EPS production by *B. licheniformis* in the presence of *K. salsicia*, which correlates with the improved survival of *B. licheniformis* in the BL-ML, BL-SR, and BL-KS biofilm combinations, and the highest survival in the BL-ML-KS combination. The EPS forms a barrier that has been reported to hamper the treatment of biofilms with conventional antibiotics by either slowing down diffusion or inactivating the antibiotics [50,51]. Our study further confirms that protection from C&D is also a community-specific behaviour, and it varies once a community changes from dual-species or trios to a more complex community.

## 5 Conclusion

In conclusion, the critical role of *K. salsicia* in promoting the growth of neighbouring bacterial species underscores the complex interspecies interactions that drive advantageous growth in bacterial biofilm communities. Furthermore, this finding highlights the potential evolutionary trade-offs that organisms may encounter in intricate community environments. In addition, considering pairwise interactions as the basic unit of higher-order effects does not adequately capture the dynamics of interactions in complex community contexts. The interactions among bacteria may significantly influence their response to C&D regimes in industrial settings, due to their spatial organization and potentially due to their EPS production. This study indicates that the presence of *K. salsicia* in the food industry is a concern due to its role in enhancing the growth of species like *S. rhizophila*, *M. lacticum*, and *B. licheniformis*. Furthermore, the post-C&D survival of these bacteria is linked to the presence of *S. rhizophila*. This highlights the importance of considering species-specific interactions when developing C&D strategies aimed at effectively eradicating biofilms in any industrial setting.

## Disclaimer

The results and conclusions in this article reflect only the authors’ view. The funding Research Executive Agency (REA), delegated by the European Commission, is not responsible for any use that may be made of the information it contains.

## CRediT authorship contribution statement

**Faizan Ahmed Sadiq:** Writing – original draft, Visualization, Validation, Software, Methodology, Funding acquisition, Formal analysis, Data curation, Conceptualization. **Koen De Reu:** Writing – review & editing, Validation, Supervision, Funding acquisition, Conceptualization. **Nan Yang:** Writing – review & editing, Visualization, Validation, Software, Methodology. **Mette Burmølle:** Writing – review & editing, Visualization, Validation, Supervision, Methodology, Conceptualization. **Marc Heyndrickx:** Writing – review & editing, Validation, Supervision, Resources, Methodology, Funding acquisition, Conceptualization.

## Declaration of competing interest

The authors declare the following financial interests/personal relationships which may be considered as potential competing interests:

Faizan Ahmed Sadiq reports financial support was provided by European Commission. If there are other authors, they declare that they have no known competing financial interests or personal relationships that could have appeared to influence the work reported in this paper.

## Data Availability

Data will be made available on request.
