# Supplementary material for: Interspecies interactions in dairy biofilms drive community structure and response against cleaning and disinfection
Source: Biofilm. 2024 Apr 8;7:100195. doi: 10.1016/j.bioflm.2024.100195 (PMC11024912; doi:10.1016/j.bioflm.2024.100195)
Supplement: Multimedia component 1 [file mmc1.docx]

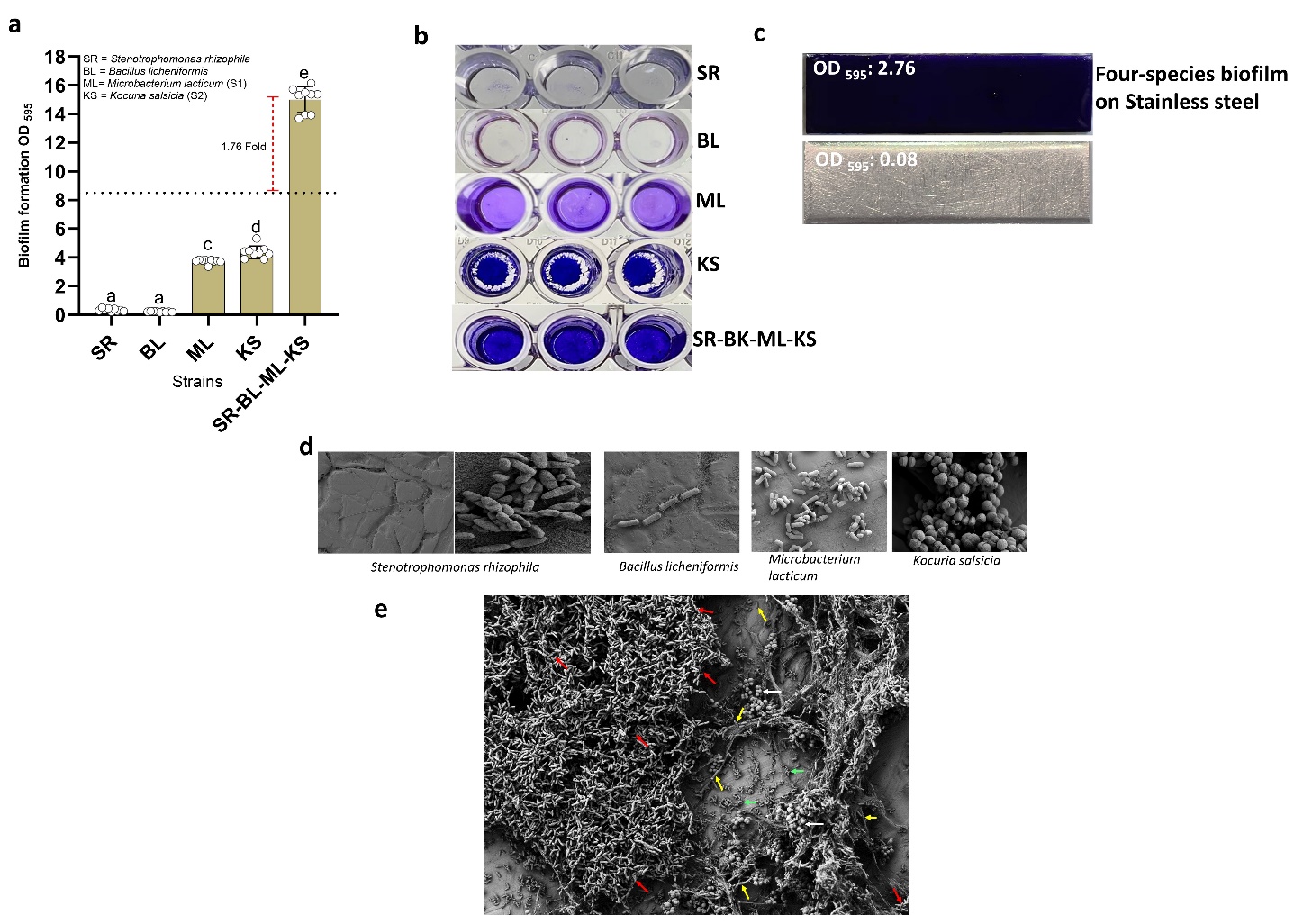


**Figure S1**. Part a shows synergistic interactions in terms of biofilm mass between *Stenotrophomonas rhizophila* (SR), *Bacillus licheniformis* (BL), *Microbacterium lacticum* (ML), and *Kocuria salsicia* (KS) in a four-species biofilm on stainless steel coupons (AISI 304 grade) in brain-heart-infusion broth. The biofilm mass of the four-species biofilm was 1.76-fold higher than the sum of biofilm masses of each of the four species in monoculture biofilms. Statistical significance was determined by one-way analysis of variance (ANOVA) followed by a Duncan's Multiple Range Test using SPSS v.23. Parts (b) and (c) show images of the crystal violet-stained biofilm matrix and cells on the surface of 96-well microtiter plates and stainless steel coupons, respectively. The optical density (OD_595_) value of the crystal violet-stained biofilm matrix and cells on stainless coupons is also indicated. Part d shows images of monoculture biofilms for each of the four strains using scanning electron microscopy (SEM). Monoculture biofilm images of SR, BL, and ML have been obtained from another experimental batch and these images were first published in our previous publication as a supplementary material (Sadiq et al., 2023a). For *S. rhizophila* only a few damaged cell could be seen on the surface, whereas for *C. indicus* no cells could be seen in monoculture. Part e shows mixed-species biofilm of the four species where cells of SR, BL, ML and KS are indicated by red, yellow, green and white arrows, respectively. *B. licheniformis* spores are also highlighted by a yellow circle.


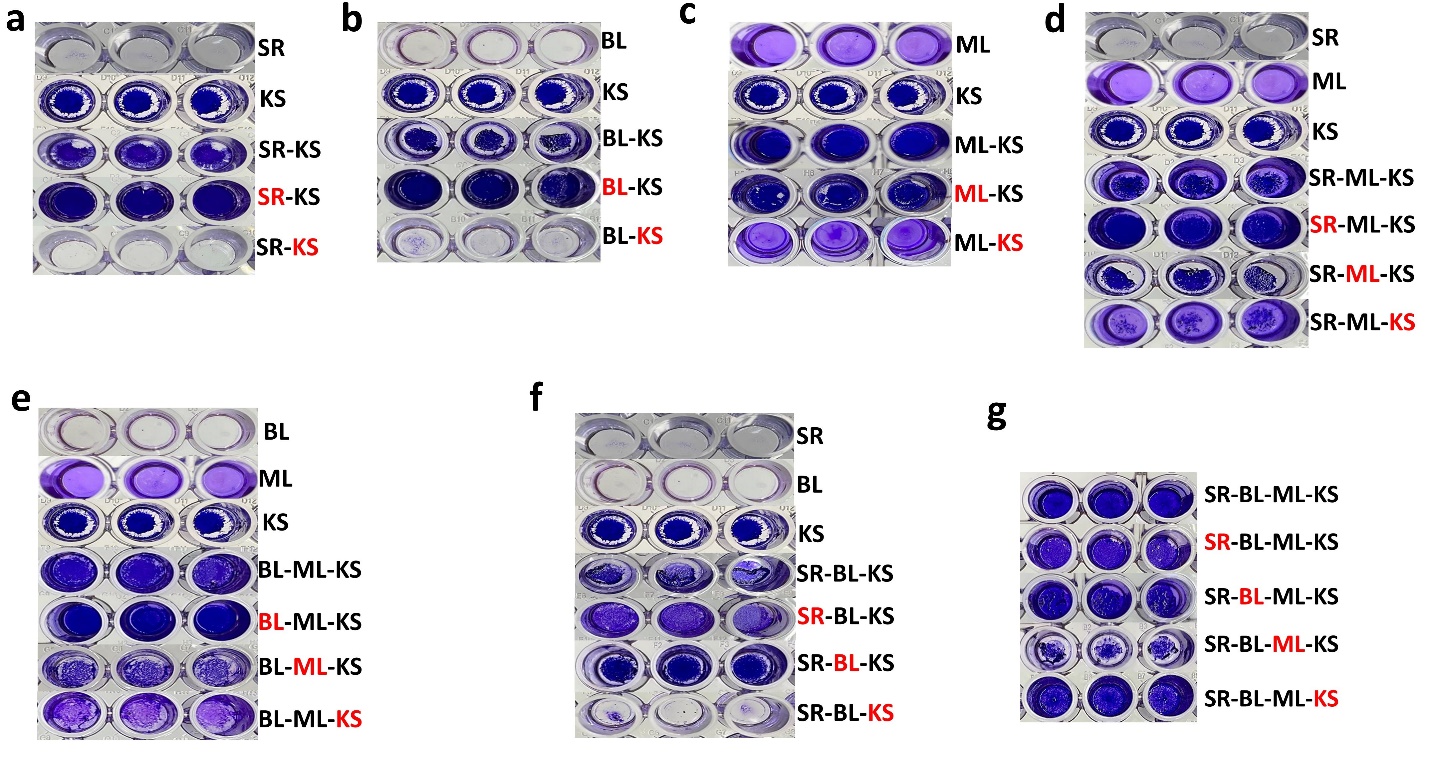


**Figure S2.** Crystal violet stained wells and biofilm mass photographed for visual representation of biofilm formation by *Stenotrophomonas rhizophila* (SR), *Bacillus licheniformis* (BL), *Microbacterium lacticum* (ML), and *Kocuria salsicia* (KS) in monoculture and different mixed-culture biofilm combinations. The influence of replacing each strain one-by-one by its cell-free-supernatant (CFS) in different mixed biofilm combinations is also shown. Strain’s abbreviation written in red indicate presence of CFS of the strain rather than its presence in viable form.


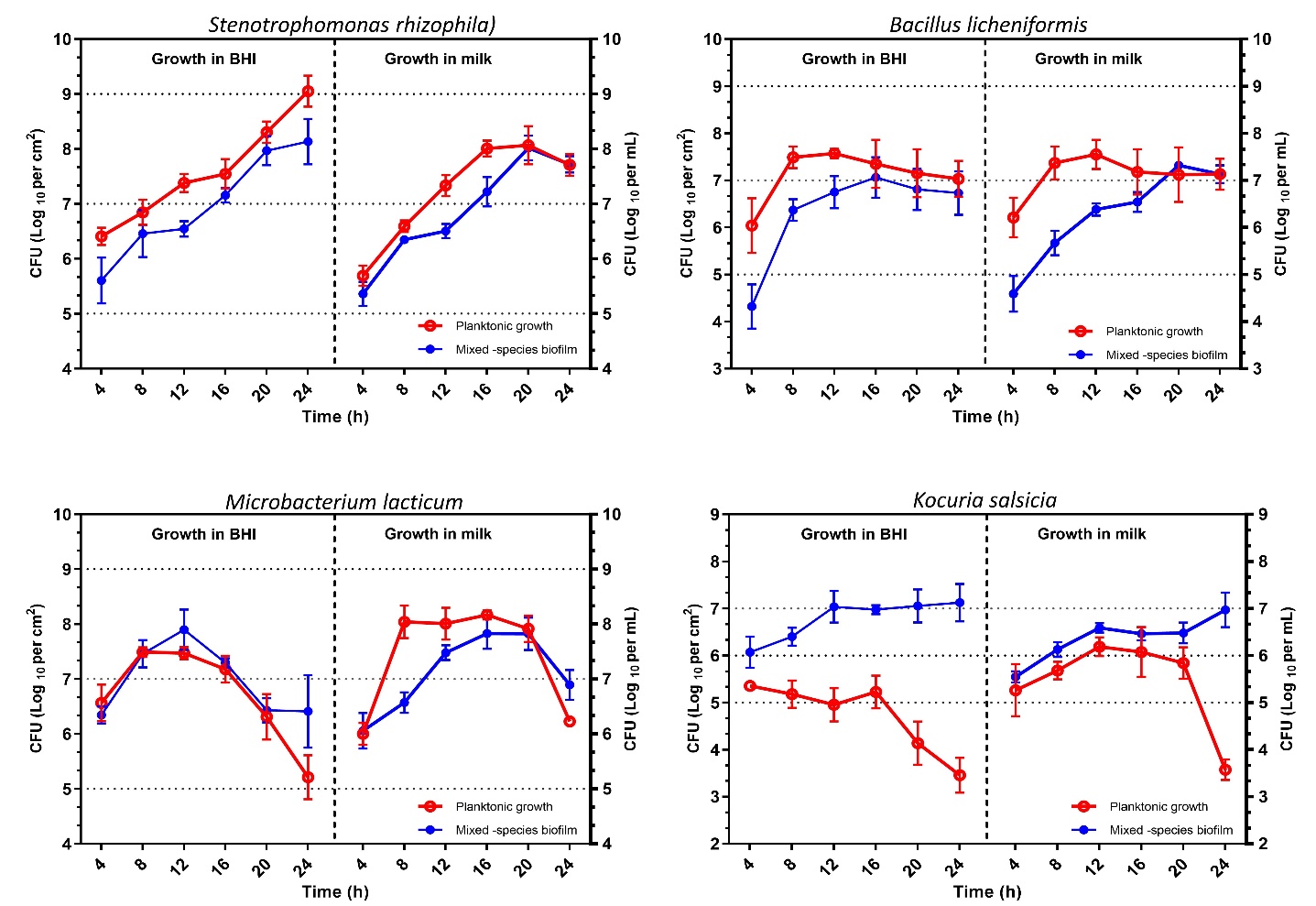


**Figure S3**. Quantitative analysis of biofilm-associated (blue) and planktonic (red) cell counts of each species in a four-species biofilm community on AISI 304 grade stainless steel coupons, cultured in skim milk and brain-heart-infusion medium. The graph shows the estimated cell numbers per cm² in biofilms and per mL in the planktonic fraction for *Stenotrophomonas rhizophila*, *Bacillus licheniformis*, *Microbacterium lacticum*, and *Kocuria salsicia*. Cell counts were determined at six time points (4, 8, 12, 16, 20, and 24 h post-coincubation) using selective media plates. Each data point is the mean of three replicates, with vertical lines representing the standard deviation.


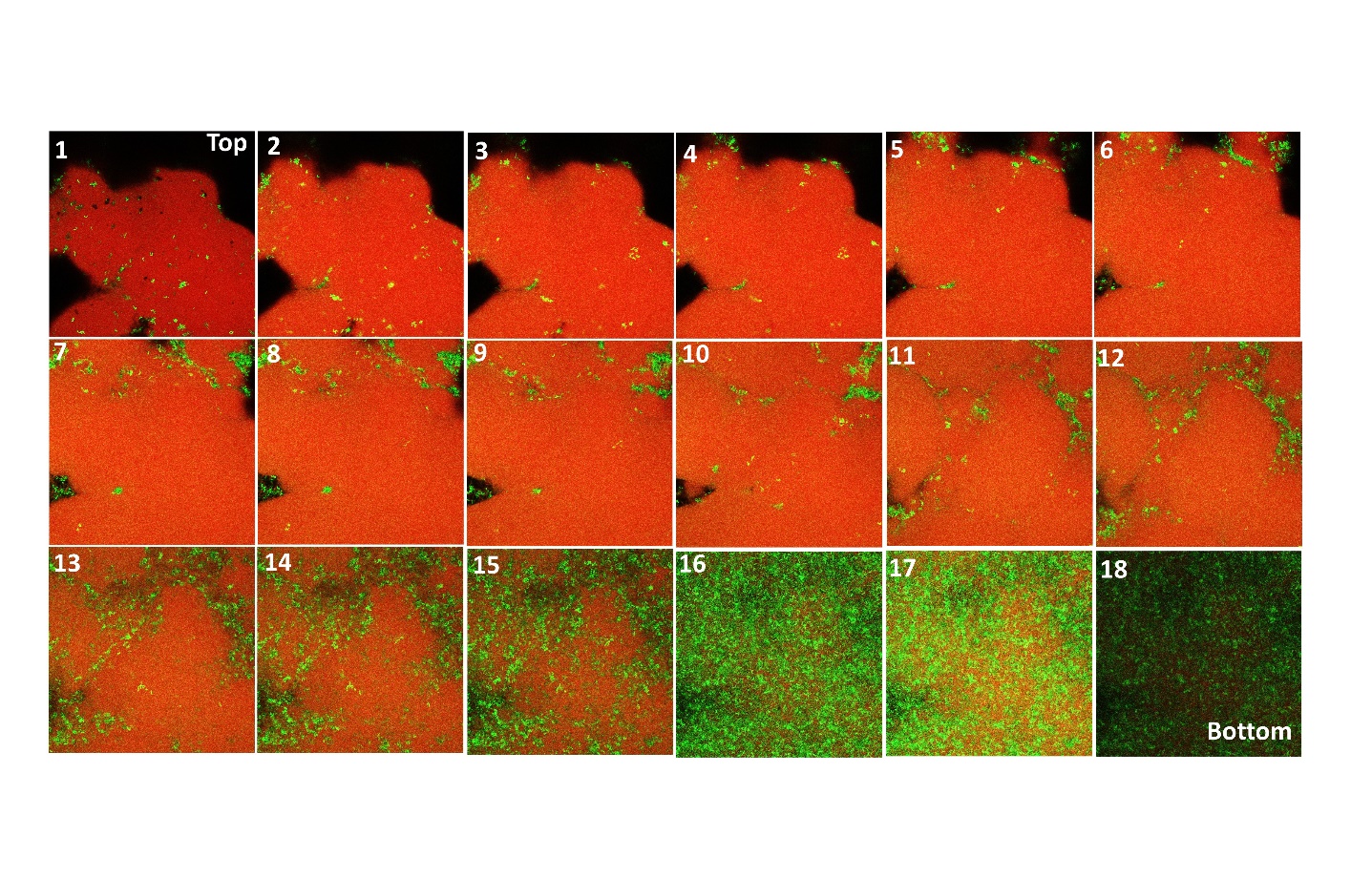


**Figure S4**. Confocal microscopy images representing sequential cross-sectional layers of a dual-species biofilm formed by *Microbacterium lacticum* (green) and *Stenotrophomonas rhizophila* (red) on a plastic coupon in brain-heart-infusion medium. Images 1-18 show progressive sections from the top to the bottom of the biofilm, with section 1 at the topmost layer and section 18 at the bottommost. *M. lacticum* can be seen as present predominantly at the base (sections 17-18), while *S. rhizophila* appears to progressively colonize the upper layers (sections 1-16), demonstrating a stratified biofilm architecture. *S. rhizophila* becomes increasingly predominant towards the top, forming a biofilm layer directly over *M. lacticum*, as observed in the upper sections.


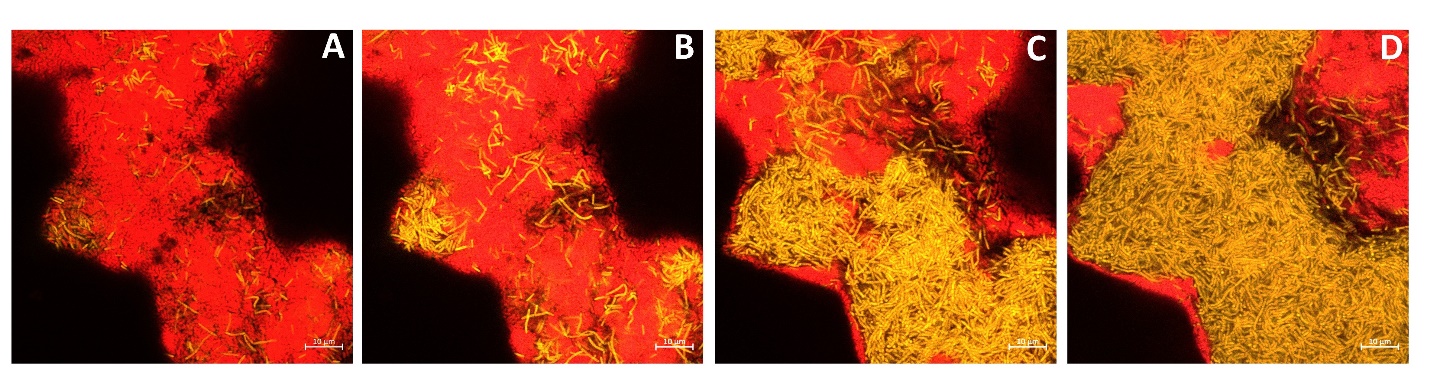


**Figure S5**. Confocal laser scanning microscopy images of a dual-species biofilm featuring *Bacillus licheniformis* (yellow) and *Stenotrophomonas rhizophila* (red). Panel A shows the topmost layer where *S. rhizophila* is the dominant species, forming an extensive layer over *B. licheniformis*. Panels B to D provide a descending sequence of cross-sectional views from the top (B) to the bottom (D), with *B. licheniformis* becoming increasingly prominent towards the bottom. In Panel D, *B. licheniformis* appears to be the dominant species at the surface, whereas the dominance of *S. rhizophila* is evident in the upper layers, particularly in Panel A.


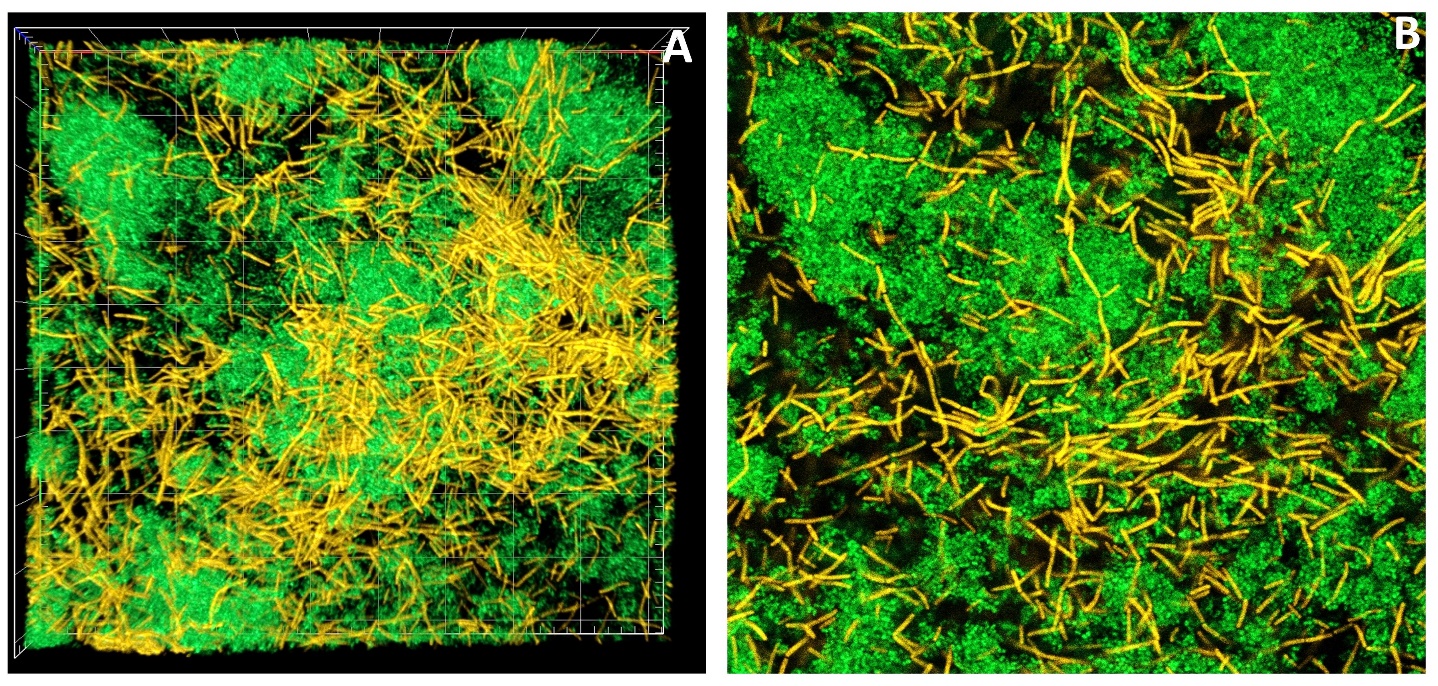


**Figure S6**. Confocal microscopy images depicting the distribution and interaction of *Bacillus licheniformis* (yellow) and *Microbacterium lacticum* (green) in a dual-species biofilm on plastic. The two images show a composite top and bottom view, highlighting the dense intertwining and layering of the two species. Panels A and B, respectively, show the bottom and top of the dual-species biofilm.
