# Supplementary material for: Interspecies interactions in dairy biofilms drive community structure and response against cleaning and disinfection
Source: Biofilm. 2024 Apr 8;7:100195. doi: 10.1016/j.bioflm.2024.100195 (PMC11024912; doi:10.1016/j.bioflm.2024.100195)
Supplement: Multimedia component 2 [file mmc2.docx]

**Supplementary Material File S1**

**Table S1**. Sequences of the oligonucleotide (oligo) probes for fluorescent *in situ* hybridization (FISH) analysis.

| **Name of the species** | **Sequences** | **Max. excitation** | **Max. emission** | **Fluorophores** |
| --- | --- | --- | --- | --- |
| *S. rhizophila* | GGGCCTTTACCCCGCCA | 649 nm | 670 nm | Cy5 |
| *B. licheniformis* | ACCGCCTGCGCGCGCTT | 550 nm | 570 nm | Cy3 |
| *M. lacticum* | CCCCACCCTTTCGCTCC | 495 nm | 520 nm | FAM |

1. **Species-specific growth media plates for selective counting**

**2.1 Antibiotic concentration**

| **Name of the antibiotic** | **Stock concentration** |
| --- | --- |
| Ciprofloxacine | 0.0004 g or 0.4 mg / mL into 1 M NaOH |
| Polymyxin B sulfate | 1 mg/ mL water |
| Colistine | 1.5 mg / mL water |
| Kanamycin A | 2.5 mg / mL water |

**2.2 Antibiotic concentration in growth media (Brain-heart-infusion medium)**

| **Name of the species** | **Ciprofloxacine** | **Polymyxin B sulfate** | **Colistine** | **Kanamycin A** |
| --- | --- | --- | --- | --- |
| *Microbacterium lacticum* | 13 µl/100 mL media | 110 µl/100 mL media | 33 µl/100 mL media | 40 µl/100 mL media |
| *Calidifontibacter indicus* | 22 µl/100 mL media | 110 µl/100 mL media | **-** | **-** |

***Stenotrophomonas rhizophila* on MacConkey agar No.3 (ThermoFisher Scientific) without antibiotics**

***Bacillus licheniformis* on nutrient agar (ThermoFisher Scientific) without any antibiotic**
